# Early risk factors for acute chest syndrome in sickle cell anemia: A pediatric study

**DOI:** 10.1371/journal.pone.0336567

**Published:** 2025-11-14

**Authors:** Mohamed Condé, Florie Bouvier, Roselyne Brat, Salim Ferrani, Régis Hankard, Philippe Connes, Georges Dimitrov

**Affiliations:** 1 Department of Pediatrics, University Hospital Centre of Orléans, Orléans, France; 2 Department of Statistics, University Hospital Centre of Orléans, Orléans, France; 3 Department of Pediatric Intensive Care, University Hospital Centre of Orléans, Orléans, France; 4 Emergency Pediatric Department, University Hospital Centre of Orléans, Orléans, France; 5 Laboratoire Interuniversitaire de Biologie de la Motricité (LIBM) EA7424, Vascular Biology and Red Blood Cell Team, Université Claude Bernard Lyon 1, Université de Lyon, Lyon, France; 6 Laboratoire d’Excellence du Globule Rouge (Labex GR-Ex), PRES Sorbonne, Paris, France; 7 Department of Pediatric Surgery, University Hospital Centre of Orléans, Orléans, France; Makerere University / Mulago National Referral Hospital, UGANDA

## Abstract

Acute chest syndrome (ACS) is a life-threatening complication of sickle cell anemia (SCA). Most often, ACS occurs during the progression of a painful vaso-occlusive crisis (VOC) in vulnerable patients. The present study aimed to identify early risk factors for ACS progression, focusing on patient assessments in a pediatric Emergency Department. In this study (2016–2022) concerning exclusively the SS and Sβ^0^ sickle cell disease genotypes, severe VOC encounters progressing to ACS were compared to uncomplicated severe VOCs. Medical history, clinical and laboratory data were collected for both groups. Out of 280 severe VOC encounters without initial respiratory symptoms, 40 progressed to life-threatening acute chest syndrome. The forty ACS (age 8.5 ± 4.3 years, 37% females) were compared with 240 severe VOCs (9.3 ± 4.4 years, 46% females). ACS was positively correlated with the occurrence of VOC at night, diffuse (multifocal) pain and increased C-Reactive protein (p < 0.05). The multivariable modelling, using generalized linear mixed-effects models, defined three risk factors for ACS occurrence: diffuse pain, night-time pain occurrence, and increased C-Reactive protein (p < 0.01). Increased use of opiates in the Emergency Department, elevated total leucocyte count, breath rate, and decreased red blood cell count were not significantly associated with ACS occurrence (p > 0.05). The initial evaluation of SCA patients’ acute pain in the Emergency Department is crucial for subsequent management during hospitalization.

## Introduction

Sickle cell anemia (SCA) is one of the most frequent monogenetic diseases worldwide, affecting more than 300,000 newborns from Africa and the Middle East [1,2]. SCA is caused by a DNA base-pair modification of the β−globin gene leading to production of the abnormal hemoglobin (Hb) HbS [3,4]. HbS may polymerize under deoxygenation, causing a mechanical distortion of red blood cells (RBCs). RBCs from SCA patients are poorly deformable and fragile, resulting in the occurrence of vaso-occlusive-like events and chronic hemolytic anemia. SCA affects over 20,000 patients in France, where it is the most common rare disease [5,6]. Symptomatic since the age of a few months, SCA causes multiorgan damage – lung, bones, retina, kidneys, brain, and heart – and reduces life expectancy by more than 20 years [7,8].

Pediatric symptomatology is largely dominated by acute and severe pain crises (vaso-occlusive crises; VOCs) requiring hospitalizations of 4–7 days for analgesic treatment by intravenous opiates associated with hydration [4,9]. Nevertheless, initial painful VOC may sometimes progress to the pulmonary complication of acute chest syndrome (ACS), a severe respiratory distress requiring hospitalization in an intensive care unit [10]. In the intensive care unit (ICU), therapy consists in decreasing the proportion of HbS in circulation via simple transfusion or exchange transfusion [11], associated with supportive respiratory care and probabilistic antibiotics in the event of fever. In rare cases, ACS progresses to multiorgan failure syndrome affecting the kidney, liver, or brain function in addition to respiratory failure. The factors that influence the progression of VOC pain to ACS are incompletely understood. The purpose of our study was to conduct a retrospective analysis of the initial clinical and laboratory data of patients with SCA who presented ACS during VOC progression in order to point out risk factors and thus optimize the monitoring and treatment of these vulnerable patients.

## Patients and methods

### Study design

We performed an Institutional Review Board-approved retrospective cohort study (IRB n° 2024 099; Ethics Committee of University Hospital of Tours, France; data accessibility for research purposes: from December 20, 2024) of the medical history of patients with severe VOCs complicated by ACS or uncomplicated VOCs hospitalized in the Pediatrics and Pediatric Intensive Care Unit of Orleans Hospital in France during a 7-year period (2016–2022). We were able to identify individual participants during data collection, but not after the data had been collected. The study was performed following relevant guidelines and regulations in accordance with the Declaration of Helsinki. Informed consent was obtained from all participants and/or their legal guardians.

ACS was defined as the appearance of new radiologic infiltrate associated with one or more clinical symptoms such as chest pain, respiratory distress, fever and cough [10].

Inclusion criteria for the study were: (a) diagnosis of SCA, (b) hospitalization for one or more severe VOCs, (c) age ≥ 1-year, (d) absence of ACS on arrival in the Emergency Department (ED) and (e) use of parenteral opiates during hospitalization. Exclusion criteria were (a) acute pain not due to VOC (viral infection such as gastroenteritis, rhinopharyngitis, angina; cholecystitis; pancreatitis), or a psychological disorder such as anxiety, (b) primary ACS diagnosed in ED, (c) respiratory signs without initial chest X-ray, (d) RBC transfusion and/or exchange transfusion (ET) during the 6 weeks preceding the ED consultation, (e) follow-up in another hospital, (f) secondary transfer from another hospital, (e) age < 1 year, (f) discharge within 24 hours of admission, and (g) refusal to participate. Each patient was seen one or more times, and each ED encounter was analyzed separately.

Demographic characteristics, nutritional parameters according to World Health Organisation standards [12], clinical history, vital and laboratory parameters from the ED were collected, analyzed, and compared between the ACS group and the VOC group. Baseline Hb was defined as the mean value of four Hb measurements at steady state. Baseline fetal Hb (HbF) was the last value of HbF of the six preceding months, usually obtained at steady state. Hydroxyurea (HU) maximum tolerated dose (MTD) was defined as the dosage at which the baseline control blood count showed a neutrophil count of around 2,000/mm^3^, without falling below 1,000/mm^3^, which is generally reached with a dosage of 20–30 mg/kg/day of HU according to the French Recommendation 2024 for pediatric SCD [13]. Night-time VOC occurrence was defined as the interval of time between 9 p.m. and 7 a.m. Location of acute pain was described as either localized (chest, abdomen, back, upper and lower limb(s), upper limb(s), lower limb(s)) or diffuse (defined as pain having two or more locations, one of which the trunk). Intensity of pain was evaluated by numerical score (0: no pain; 10: maximum pain) if age ≥ 7years, or EVENDOL score [14] if age < 7years.

### Statistical analysis

Baseline characteristics were summarized using counts and percentages for categorical variables, median with interquartile range or mean with standard deviation for continuous variables. The distributions of continuous variables were assessed with the Shapiro–Wilk test. Univariable comparisons were first performed between ACS and VOC groups using mixed-effects models to account for repeated measures per patient. Additional univariable comparisons were then performed across hydroxyurea groups (“MTD”, “Below MTD”, “Not on HU”), with likelihood ratio tests from generalized linear mixed models to account for repeated measures per patient, while continuous variables were summarized as the median [25th–75th percentile] and compared using ANOVA. Post-hoc pairwise comparisons were performed when the overall test was significant. Variables with p < 0.2 in univariable analyses were considered for multivariable modeling using generalized linear mixed-effects models, with variable selection guided by Akaike and Bayesian Information Criteria (AIC, BIC). All analyses were performed in R (Version 4.4.2).

## Results

### Population

**Fig 1:**
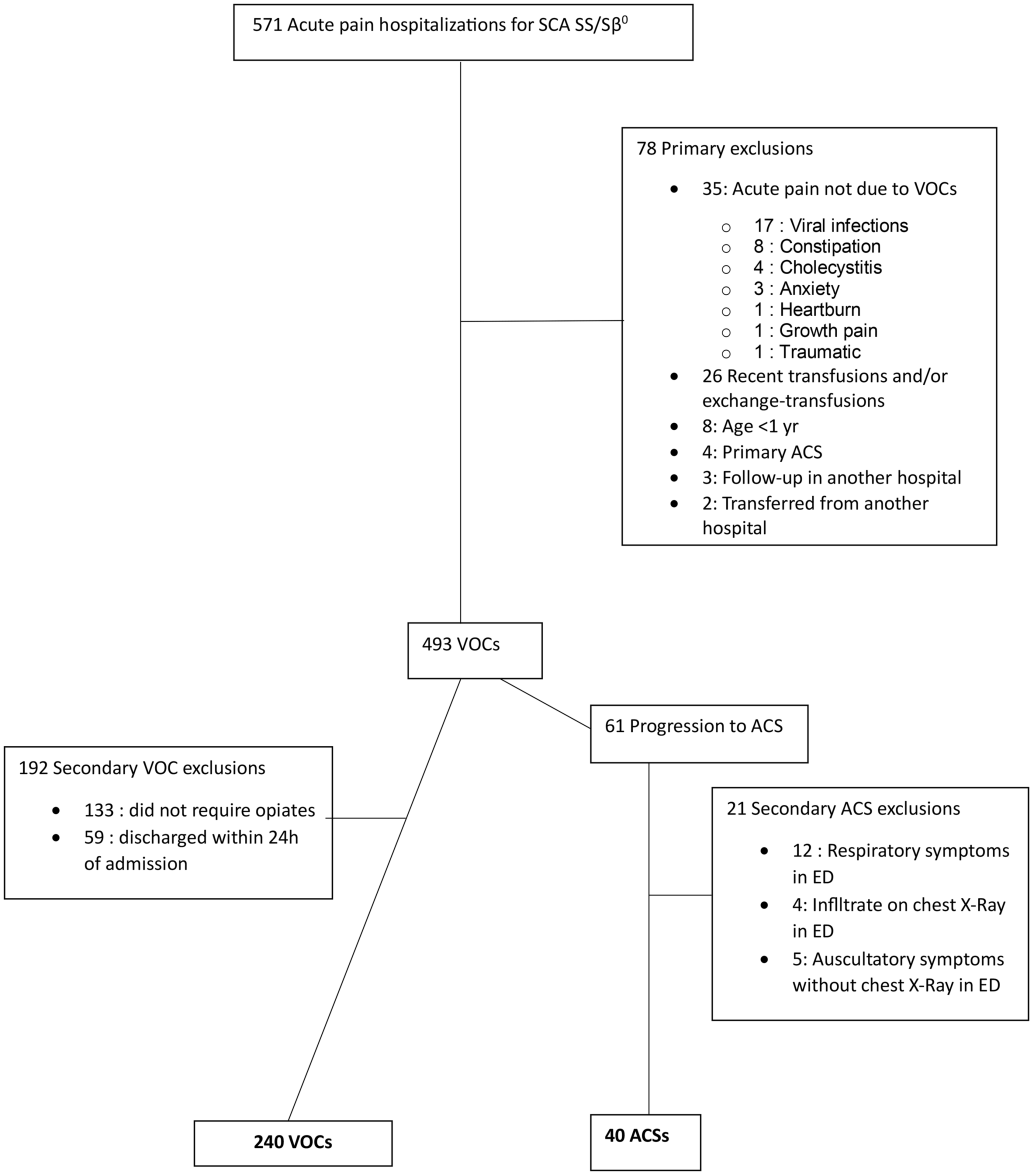
Flow chart of VOC encounters during the 7-year period (2016–2022).

We reviewed a total of 571 charts over a 7-year period (from January 2016 through December 2022). Among these, 280 encounters in 70 patients were included in our analysis based on our eligibility criteria (Fig 1).

Among the included patients, twenty-eight (40%) were female, six (8.57%) had the Sβ0 genotype, sixty-four (91.43%) had the SS genotype, and the average annual number of severe VOCs was 0.8 (S1 Data). The number of encounters for each patient ranged from 1 to 15 during the study period. Twenty-three patients were included once, and 47 more than once. Twenty-nine patients developed 40 ACSs: twenty-one patients had only one ACS, five patients had two ACSs, and three patients had three ACSs. Of the patients who developed ACS, all but four had at least 1 VOC (and up to 14 VOC) during their other hospitalizations. Thus, forty ACS encounters were compared with two hundred forty severe VOC encounters.

### Between-group results and comparative analysis

#### Characteristics at inclusion.

Characteristics of the 280 VOCs at inclusion were similar between the two groups except for the HU dosage and the HU maximum tolerated dose (Table 1 and S2 Data).

**Table 1:** Characteristics of the 280 VOCs at inclusion.

	VOC	ACS	p-value
Number	240 (85.71%)	40 (14.29%)	
Age, yr^a^	9.3 (4.4)	8.5 (4.3)	0.26
Females	111 (46.25%)	15 (37.50%)	0.43
SCD genotype SS/Sβ^0^	216 (90%)/ 24 (10%)	34 (85%)/6 (15%)	0.36
Baseline Hb^b^, g/dL	8.41 (0.95)	8.53 (0.84)	0.42
Baseline HbF^c^, %	11.59 (11.80)	11.07 (5.26)	0.75
G6PD deficiency	27 (11.25%)	7 (17.50%)	0.30
Nutritional status			
BMI, percentiles for age	36.56 (28.88)	34.73 (26.05)	0.87
BMI < 5th percentile	37 (15.42%)	2 (5.00%)	0.07
BMI > 85th percentile	16 (6.67%)	3 (7.50%)	0.74
Ratio Weight/ Expected Weight for Age, %	96.4 (18.8)	95.6 (21.1)	0.97
Ratio Weight/ Expected weight for Height, %	96.9 (13.36)	95.9 (15.1)	0.87
Ratio Height/ Expected height for Age, %	99.9 (5.1)	99.7 (4.3)	0.91
Hydroxyurea treatment	144 (60.00%)	26 (65.00%)	0.55
Hydroxyurea dosage, mg/kg/d	20.5 (4.59)	23.4 (4.56)	**0.01**
Hydroxyurea Maximum tolerated dose	63 (43.75%)	19 (73.08%)	**0.04**
History of chronic monthly exchange transfusion	17 (7.08%)	2 (5.00%)	0.75
SCD history			
Transfusion during last year	85 (35.42%)	12 (30.00%)	0.59
Exchange transfusion during last year	34 (14.17%)	7 (17.50%)	0.89
ICU^d^ admission during the last year	53 (22.08%)	9 (22.50%)	0.63
0 VOC hospitalizations/year*	82 (34.17%)	19 (47.50%)	0.23
1–2 VOC hospitalizations/ year*	104 (43.33%)	14 (35.00%)	0.52
More than 2 VOC hospitalizations/year*	54 (22.78%)	7 (17.50%)	0.53
Number of previous ACSs	0.5 (0.87)	0.7 (0.92)	0.86
Cerebral vasculopathy	16 (6.67%)	5 (12.50%)	0.22
Osteonecrosis	6 (2.50%)	1 (2.50%)	0.72
Splenectomy	23 (9.58%)	4 (10.00%)	0.62
Heart involvement (dilated cardiomyopathy)	17 (7.08%)	3 (7.50%)	0.91
Retinopathy	26 (14.77%)	5 (14.71%)	0.25
Asthma	31 (12.97%)	5 (12.50%)	0.75
Cholelithiasis	89 (37.08%)	13 (32.50%)	0.82
Bacterial infection precipitated by SCD			0.05
Salmonella Typhimurium (osteomyelitis)	20 (64.52%)	0 (0%)	
Streptococcus pneumoniae (meningitis)	11 (35.48%)	1 (100%)	

Abbreviations and legend: ^a^years, ^b^hemoglobin, ^c^fetal hemoglobin; ^d^Intensive Care Unit; *: include only severe VOC hospitalizations i.e. VOCs requiring opiates; (): standard deviations unless otherwise indicated; p-values were derived from univariable mixed-effects logistic regression models with ACS occurrence (yes/no) as the outcome and patient as a random effect.

Steady-state characteristics of SCA (baseline Hb, baseline HbF), nutritional status parameters, and clinical history did not differ between the ACS and VOC groups. There was no history of hepatic and kidney involvement. The mean age at the first ACS of the eight patients with recurrent ACS was 5.7 ± 3.5 years.

Comparison between the three HU usage groups - “MTD”, “Below MTD”, “Not on HU” – showed that VOCs with and without ACS differed significantly (S1 Table) (p = 0.04). For laboratory parameters, neutrophils (NEU) decreased progressively with increasing HU dosage: medians were 9.25 [6.37–11.70] in “Not on HU”, 7.72 [5.04–9.99] in “Below MTD”, and 5.09 [3.83–7.29] in “MTD” (p < 0.0001). Mixed-model estimated marginal means confirmed significant pairwise differences between all groups (Not on HU vs. Below MTD: p = 0.011; Not on HU vs. MTD: p < 0.0001; Below MTD vs. MTD: p = 0.0046). Similarly, mean corpuscular volume (MCV) increased with HU dosage: 76.0 [70.5–83.0] in “Not on HU”, 84.9 [75.8–92.0] in “Below MTD”, and 91.5 [83.0–95.8] in “MTD” (p < 0.0001). Adjusted means showed the same stepwise increase (77.1, 85.4, and 89.4 fL, respectively), with all pairwise contrasts being highly significant (p ≤ 0.0001).

In contrast, baseline HbF%, baseline hemoglobin, and ΔHb did not differ significantly across HU groups (all p ≥ 0.22). Recurrent ACS encounters were treated by HU on MTD. Only one patient who presented with two ACS at 1.3 and 1.7 years did not receive HU because of his young age. HU indications in the VOC group and the ACS group were repeated VOCs (64% versus 58%), previous ACS (14% versus 19%), baseline Hb < 8g/dL (1% versus 0%), and baseline Hb < 8g/dL associated to repeated VOCs (20% versus 23%).

#### Clinical and laboratory parameters in the Emergency Department.

The median VOC-to-hospitalization interval (Table 2) was approximately 12 hours for both groups. Night-time VOC occurrence was significantly higher in the ACS versus the VOC group (p = 0.01).

**Table 2:** Clinical and laboratory characteristics at the Emergency Department presentation, stratified by ACS occurrence.

	VOC 240 (85.71%)	ACS 40 (14.29%)	p-value	Missing
VOC-onset-to-hospitalization interval (h)	12 [7, 24]	11.5 [6.8, 24.5]	0.96	0
Night-time VOC occurrence	59 (24.69%)	18 (45.00%)	**0.01**	1
Transcutaneous O2 saturation (%)	99 [98, 100]	100 [98, 100]	0.11	0
Breaths/min	23 [21, 25]	24 [21.5, 28]	0.17	1
Systolic blood pressure (mm Hg)	117 [108, 125]	118 [109, 126]	0.74	26
Diastolic blood pressure (mm Hg)	73 [65, 79]	72 [62, 80]	0.57	26
Heart rate at admission	98 [88, 113]	98.5 [89, 108]	0.79	11
Temperature (°C)	36.8 [36.5, 37.2]	36.8 [36.6, 37.2]	0.89	12
Location of acute pain				
Diffuse	70 (29.17%)	21 (52.50%)	**<0.01**	0
Chest	20 (8.33%)	3 (7.50%)	0.96	0
Abdominal	44 (18.33%)	11 (27.50%)	0.20	0
Back	23 (9.58%)	3 (7.50%)	0.56	0
Upper and lower limb(s)	15 (6.25%)	1 (2.50%)	0.30	0
Upper limb(s)	29 (12.08%)	1 (2.50%)	0.08	0
Lower limb(s)	39 (16.25%)	0 (0%)	**1**	0
Pain rating				5
Numeric pain rating scale at admission	8 [6, 9]	8 [7, 9]	0.22	0
EVENDOL pain rating scale at admission	6 [4, 8]	7 [6, 9]	0.12	5
Opiate use in ED	182 (75.83%)	36 (90.00%)	0.05	0
TLC^a^ (x10^3^/mm^3^)	12.6 [9.7, 16.5]	14.65 [11.03, 17.05]	0.09	0
NEU^b^ (x10^3^/mm^3^)	7.51 [4.82, 10.32]	7.82 [5.89, 10.86]	0.13	0
LYM^c^ (x10^3^/mm^3^)	3.03 [2.09, 4.87]	3.35 [2.32, 4.12]	0.72	0
MO^d^ (x10^3^/mm^3^)	1.08 [0.72, 1.63]	1.28 [0.85, 1.77]	0.76	0
RBC^e^ (x10^6^/mm^3^)	3.04 [2.73, 3.88]	3 [2.83, 3.74]	0.18	0
Hct^f^ (%)	26 [23.17, 28.22]	26.1 [23.87, 28.63]	0.82	0
Hb^g^ (g/dL)	8.7 [7.9, 9.5]	8.8 [8, 9.43]	0.76	0
∆ Hb (g/dL)	−0.3 [−1, 0.2]	−0.15 [−0.6, 0.3]	0.26	1
MCV^h^ (fL)	82.5 [75, 91]	84 [75, 94]	0.80	0
MCH^i^ (pg/cell)	27.5 [24.4, 31.0]	27.9 [25.6, 31.3]	0.82	2
MCHC^j^ (g/dL)	33.7 [33, 34.5]	34.0 [33, 34.5]	0.66	0
RCDW^k^ (%)	20.4 [18.3, 23.3]	21.3 [19.0, 23.4]	0.24	0
PLT^l^ (x103/mm3)	317 [234.5, 396.3]	288 [232.8, 370.5]	0.39	0
RC^m^ (x103/mm3)	234.4 [171.0, 285.6]	234.3 [192.4, 283.4]	0.78	66
CRP^n^ (mg/L)	6.1 [2.8, 13.5]	10.3 [4.6, 30.3]	**0.01**	6

All variables are presented as median [25% IQR – 75% IQR] unless otherwise specified. P-values were derived from univariable mixed-effects logistic regression models with ACS occurrence (yes/no) as the outcome and patient as a random effect. Missing data are indicated in the last column.

Abbreviations and legend: ^a^total leucocyte count; ^b^Neutrophils; ^c^Lymphocytes; ^d^Monocytes; ^e^Red Blood Cells; ^f^hematocrit; ^g^hemoglobin; ^h^mean corpuscular volume; ^i^mean corpuscular haemoglobin; ^j^mean corpuscular hemoglobin concentration; ^k^Red cell distribution width; ^l^Platelets; ^m^Reticulocytes count; ^n^C-Reactive Protein.

Median time interval of VOC-onset-to-ACS was 53.5 hours [40.8, 78.8]. Median time interval of ACS-onset since admission was 38 hours [23.8, 48.5]. None of the vital parameters (heart rate, respiratory rate, blood pressure, temperature and transcutaneous O2 saturation) differed significantly between the two groups. Retrospective analysis of potential precipitating factors over the preceding 5 days in the ACS group versus the VOC group yielded the following results: exertion in 10% and 3% respectively (p = 0.06), less oral hydration in 10% and 1.3% (p = 0.01), viral infection in 20% and 13% (p = 0.21), cold in 7.5% and 5.42% (p = 0.71), vomiting in 2.5% and 3.8% (p = 1), diarrhoea in 0% and 1% (p = 1), airplane travel in 0% and 1%, and dysmenorrhea in 0% and 0.4% (p = 1). In 72% and 50% of cases respectively (p = 0.01), the VOC precipitating factor remained unfound. Febrile condition (temperature ≥ 38°C) on arrival in the ED was also found in the VOC and ACS groups (10.83% and 7.5%, p = 0.78). Bacteriological explorations (blood cultures, urine analysis) in febrile patients were negative. Localized pain in the lower limb(s) was frequent in the VOC group and absent in the ACS group, while diffuse pain was significantly more frequent in the ACS group (p < 0.01). The other locations of pain were similar in the two groups (Table 2). Pain rating and opiate use in ED were not statistically different in the two groups. Complete blood count showed no difference between the two groups, but C-Reactive protein (CRP) was significantly increased in the ACS versus the VOC group (p = 0.01). The presence of nucleated red blood cells (NRBC) tended to be less frequent in the VOC group compared to the ACS group (p = 0.054). Absolute median NRBC count was not significantly different between the two groups (p = 0.119).

### General analysis

In this multivariable generalized linear mixed-effects model assessing risk factors for acute chest syndrome (ACS) during vaso-occlusive crises (VOC), several variables were identified as significant predictors (Table 3).

**Table 3:** Risk factors for ACS (multivariate analysis).

Summary of generalized linear mixed-effects model			
Variable	OR^a^	95% CI^b^	p-value
(Intercept)	0.00	0.00, 0.03	**<0.001**
Night time VOC occurrence			**0.009**
no	–	–	
yes	3.57	1.38, 9.23	
Diffuse pain			**<0.001**
no	–	–	
yes	5.69	2.06, 15.7	
Opiate use in the ED			0.066
no	–	–	
yes	3.46	0.92, 13.0	
TLC^c^	1.05	0.95, 1.15	0.32
CRP^d^	1.02	1.01, 1.04	**0.006**
Breaths/min	1.02	0.92, 1.14	0.65
RBC^e^	1.35	0.94, 1.94	0.10

Abbreviations: ^a^Odds ratio; ^b^confidence interval; ^c^total leucocytes count; ^d^C-reactive protein; ^e^red blood cells

Night time occurrence of VOC, diffuse pain and elevated CRP levels were significantly associated with a higher likelihood of developing ACS (p < 0.01). Opiate use in the ED showed a trend towards increased risk, though it did not reach statistical significance (OR 3.46, 95% CI 0.92–13.0, p = 0.066). Other parameters, including TLC, breaths/min, and RBC, were not significantly associated with ACS in this cohort. Random intercepts were included to account for multiple VOC episodes per patient, allowing the model to appropriately address intra-patient correlation.

## Discussion

The objective of the present study was to provide ED pediatricians with early predictive elements suggesting the progression of acute sickle cell pain towards a life-threatening ACS in the absence of initial respiratory signs. In current clinical practice, it is very useful to determine early whether a SCA patient presenting with a painful crisis is at risk of progressing to ACS. If a patient shows early risk factors for acute ACS, monitoring should be intensified [13]. Our results clearly suggest that the frequency of CRP measurement should be increased as CRP has been identified as a risk factor for ACS occurrence. In addition, patient pain should be assessed very regularly (particularly at night) and any change in pain location should be carefully noted. The patient should be hospitalized in the intensive care unit for continuous monitoring of vital signs, in particular gasometric signs (hypercapnia), radiological signs (alveolar-interstitial pulmonary infiltrates), and clinical signs of respiratory decompensation (tachypnea, signs of respiratory distress, desaturation). Therapeutically, it is necessary to implement multiple daily sessions of incentive spirometry and to be prepared to initiate non-invasive ventilation as well as probabilistic antibiotic therapy. Urgent exchange transfusion may be required [13].

In our cohort, almost 53% of encounters who secondarily developed ACS presented with initial diffuse (multifocal) pain and not with a localized chest pain. Lower limb pain was never associated with ACS occurrence. Patterson et al. [15] reported that chest (29%) and abdominal pain (30%) were the most frequent pain locations in 386 ACS episodes, while these locations represented 8% and 28% of our cohort, respectively. These differences could be explained by the fact that we focused on the initial pain location in ACS encounters without any respiratory signs in the ED, whereas Patterson et al. analyzed all the ACS encounters (including those presenting with respiratory signs).

Our univariable and multivariable analyses showed that ACS occurrence was associated with night-time VOC occurrence, which was not the case in adults with sickle cell disease [16]. It is possible that cold and lack of oral hydration precipitated night VOC pain. Moreover, pediatric patients receive instructions to consult in the ED as soon as pain is uncontrolled by oral grade II analgesics. Night pain is also possibly associated with anxiety, especially in patients with previous ACSs and ICU hospitalizations. Social factors, such as poorly heated accommodation, could be involved.

C-Reactive protein was significantly increased in the ACS group and was another risk factor for ACS occurrence. CRP elevation was never associated with proven bacterial infection in our cohort. CRP increase is a clear sign of systemic aseptic inflammation [17,18]. Fever, a classical sign of inflammation and/or infection, was not frequent in either the ACS or the VOC group, and all blood cultures of our patients remained negative. Alghamdi et al. [19] reported that recent upper respiratory tract symptoms before admission were predictors of developing ACS, a result not confirmed in our study. Moreover, viral prevalence in nasopharyngeal secretions was similar in both groups. Whether respiratory microorganisms, particularly frequent in young children, trigger and/or are involved in the inflammation process of ACS remains to be proven. Nevertheless, the adjusted odds ratio of “increased CRP” was rather low, suggesting the presence of inflammation in both groups.

In contrast to two previous studies [15,20], our results do not support the role of age (less than 4 years) at first ACS as a risk factor for ACS recurrence. However, the number of encounters with ACS included in our study may have been insufficient to test this hypothesis. Unlike Ballantine et al. [21], our analysis did not demonstrate that NRBC elevation predicted ACS occurrence either. Further studies with larger groups are needed to confirm the lack or presence of such associations.

The majority of encounters included in our analysis were treated by HU – 65% for the ACS group and 60% for the VOC group – mainly in order to prevent repeated VOCs and ACSs. HU dosage and percentage of encounters on HU MTD were significantly higher in the ACS group. Markers of HU intake were more evident in the ACS group (i.e., higher MCV, lower neutrophils count), even though target levels of neutrophil decrease (approximately 2,000/mm^3^) were not often reached, probably because prescribers were cautious about HU myelotoxicity. The higher HU use in the ACS group reflects the greater clinical severity of patients in this group and should not be misinterpreted as HU increasing the risk of ACS. The efficiency of HU in the prevention of recurrent VOCs and ACSs is widely recognized [22,23], and low patient adherence to HU treatment is estimated at less than 50% [24–27]. Active and repetitive therapeutic education is necessary, however, to improve HU observance in the short, medium, and long term [28,29].

The main limitation of our study is its retrospective design, which may have led to the lack of some data. For instance, we did not identify any association between asthma diagnosis and ACS occurrence, while two large cohorts of more than 3,000 participants each [30,31] demonstrated a statistically significant relationship between asthma diagnosis and the incidence of ACS. Moreover, the VOC precipitating factor remained “unfound” in more than 50% of encounters. Additionally, we had very few measures of serum lactate dehydrogenase and of liver enzyme concentrations because they were missing in patients’ files.

In conclusion, our study demonstrated that the initial evaluation of SCA children consulting for acute pain in an ED is crucial for subsequent management. VOC pain without initial respiratory symptoms, diffuse (multifocal) pain, night-time VOC occurrence, and elevated CRP are key signs suggesting unfavourable clinical progression towards a potentially life-threatening ACS.

## Supporting information

S1 Data Raw data. General characteristics of the cohort.
(XLSX)

S2 Data Raw data. Detailed characteristics of the cohort.
(XLSX)

S1 Table Patient characteristics and laboratory parameters by hydroxyurea treatment group. (DOCX)
