# Supplementary material for: Early risk factors for acute chest syndrome in sickle cell anemia: A pediatric study
Source: PLoS One. 2025 Nov 14;20(11):e0336567. doi: 10.1371/journal.pone.0336567 (PMC12617906; doi:10.1371/journal.pone.0336567)
Supplement: S1 Table — (DOCX) [file pone.0336567.s003.docx]

**S1 Table. Patient characteristics and laboratory parameters by hydroxyurea treatment group**

|  | | **On HU** | | **Not on HU**  **110 (39.29%)** | **p-value** | **Missing** |
| --- | --- | --- | --- | --- | --- | --- |
|  |  | **MTD**  **82 (29.29%)** | **Below MTD**  **88 (31.42%)** |  |  |  |
| **Group** | |  |  |  | **0.04** | 0 |
|  | VOCs w/o ACS | 63 | 81 | 96 |  |  |
|  | VOCs w/ ACS | 7 | 19 | 14 |  |  |
| **NEU^a^ (x10^3^/mm^3^)** | | 5.09 [3.83,7.29] | 7.72 [5.04,9.99] | 9.25 [6.37,11.70] | **<0.0001** | 0 |
| **MCV^b^, fL** | | 91.50 [83.00 ,95.80] | 84.90 [75.80,92.00] | 76.00 [70.50,83.00] | **<0.0001** | 0 |
| **Baseline HbF^c^, %** | | 11.30 [6.17,15.00] | 9.55 [4.60,15.20] | 9.80 [6.20,16.00] | 0.22 | 1 |
| **Baseline Hb, g/dL** | | 8.00 [7.50,9.00] | 8.70 [8.00,9.00] | 8.35 [8.00,9.00] | 0.22 | 1 |
| **∆ Hb^d^, g/dL** | | -0.20 [-0.78,0.20] | -0.20 [-1.05,0.15] | -0.30 [ -1.00,0.40] | 0.46 | 1 |

Note: All variables are presented in median [25% IQR – 75% IQR] unless otherwise specified.

Abbreviations and legend: ^a^Neutrophils; ^b^mean corpuscular volume; ^c^fetal hemoglobin; ^d^difference between baseline hemoglobin value and VOC hemoglobin value.
